# 587. Exploring Occupational/physical Therapy Utilization, Frailty, and Discharge Disposition Within Older Patients Following Burn Injury

**DOI:** 10.1093/jbcr/irag033.360

**Published:** 2026-04-06

**Authors:** David M Hill, Colette Galet, Shawn Tejiram, Kathleen S Romanowski, Mary W Boruff

**Affiliations:** Firefighters Regional One Burn Center, Memphis, TN; University of Iowa, Iowa City, IA; MedStar Washington Hospital Center, Washington, DC; Shriners Children's - Northern California, Sacramento, CA; Firefighters Regional One Burn Center, Memphis, TN

## Abstract

**Introduction:**

Following burn injury, care is tailored to each patient’s specific injury and baseline functional capabilities. Even if not formally scored, we believe frailty is currently being utilized and may be a useful tool to guide care and disposition planning.

**Methods:**

Following IRB approval, we conducted a retrospective multicenter cohort study of patients ≥60 years admitted to 12 burn centers (1/2017–12/2019). Demographics, injury characteristics, disposition, and many medical management specific variables were collected. Frailty was measured with the Canadian Study of Health and Aging Clinical Frailty Scale (1 – 7) and categorized as fit (< 4), prefrail (=4), or frail (>4). The number of occupational (OT) and physical therapy (PT) sessions totaled from individual 15-minute units. Discharge disposition was categorized 1 to 6 according to increasing necessary level of care. Death was removed and analyzed separately. There were three hypotheses explored in this analysis, focusing on the number of therapy sessions, frailty, and disposition. Univariate and multivariable analyses were performed utilizing chi-square test, Kruskal-Wallis test, and linear regression with *p*<.05 considered significant. The modified Baux score was considered, but was subsequently removed from modelling, as inhalation injury was not significantly associated with therapy sessions and age with either outcome in this older cohort.

**Results:**

Data was collected on 1632 patients. Both the number of OT and PT sessions were negatively and independently associated with increasing admission frailty. (Figure 1) These hypotheses for OT (*p*<.001) and PT (*p*=.04) remained true after controlling for a possible center effect, percent total body surface area (TBSA) burned, length of stay, and presence of a hand injury (specific for OT sessions) and when categorically condensing frailty. Frail patients had nearly a seven times higher mortality than non-frail. Frailty was positively and independently associated with discharging to a higher level of care facility (*p*<.001) after controlling for center, TBSA, and presence of inhalation injury. In separate analyses controlling for center, injury characteristics, length of stay, and admission frailty, both the number of OT and PT sessions were positively and independently associated with being discharged to a higher care facility (*p*=.048 and *p*=.007, respectively).

**Conclusions:**

Frailty scores can be utilized to plan the care of older patients admitted with burn injuries. Fit patients are more likely to return home with minimal assistance, while frail patients are more likely to be placed in a high level of care facility. Additionally, baseline frailty may be a meaningful measure to aid prognostic discussions.

**Applicability of Research to Practice:**

Frailty assessment at admission provides prognostic and care planning value and should be integrated into burn care to support patient-centered care and family discussions.

**Funding for the study:**

N/A.

